# Supplementary material for: Mechanistic insights into chromatin targeting by leukemic NUP98-PHF23 fusion
Source: Nat Commun. 2020 Jul 3;11:3339. doi: 10.1038/s41467-020-17098-4 (PMC7335091; doi:10.1038/s41467-020-17098-4)
Supplement: Supplementary file 1 — Supplementary Information [file 41467_2020_17098_MOESM1_ESM.pdf]

Supplementary Information

Mechanistic insights into chromatin targeting by leukemic NUP98-PHF23 fusion

Yi Zhang, et al.

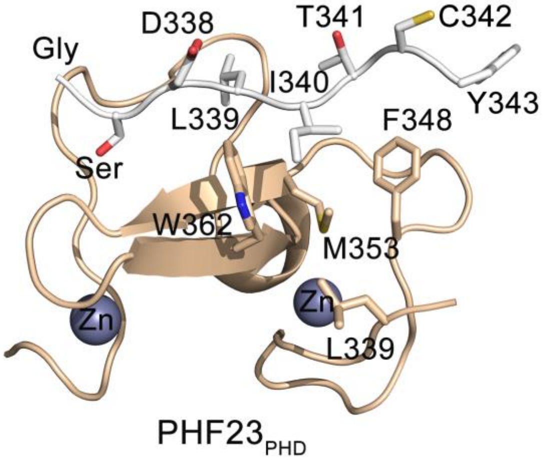

Supplementary Figure 1. Structure of $PHF23_{PHD}$. The N-terminus of another $PHF23_{PHD}$ molecule (white; labeled) binds in the histone-binding site of the domain. The side chain of I340 occupies the aromatic cage. Gly and Ser residues derived from a vector are shown and labeled. Related to Figure 2.

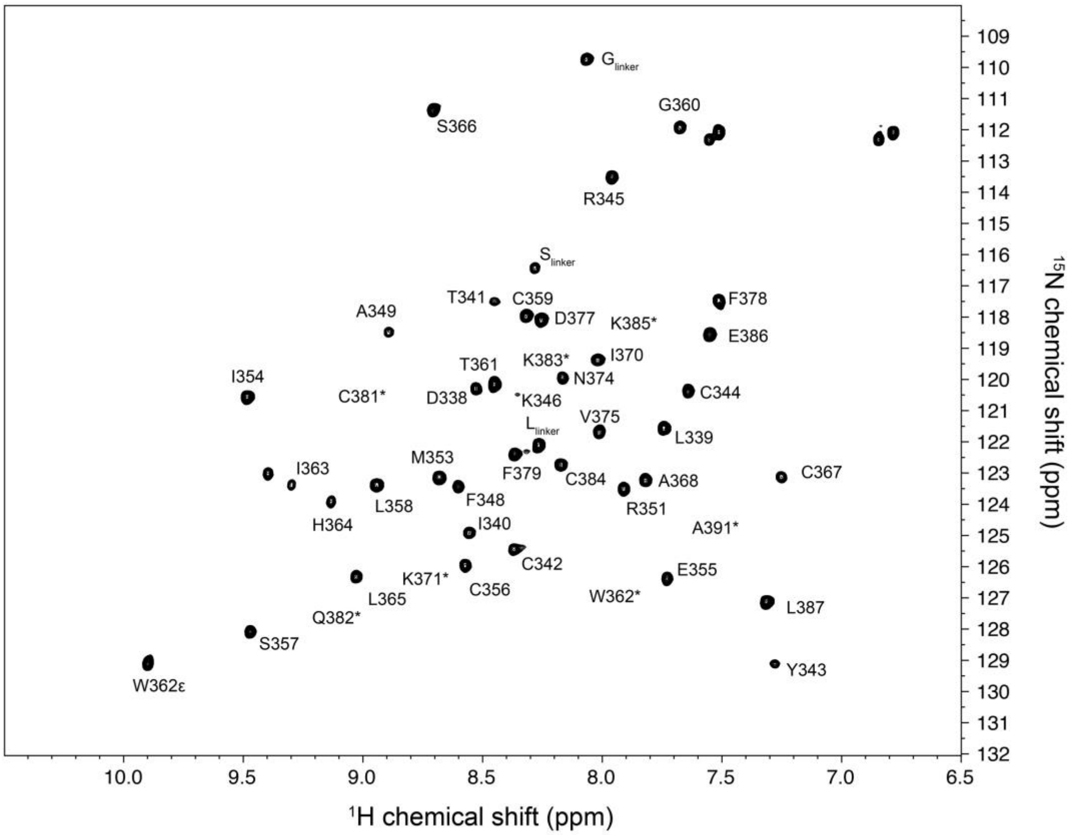

Supplementary Figure 2. Backbone assignments of PHF23$_{PHD}$. ^{1}H,^{15}N HSQC spectrum of PHF23$_{PHD}$. Backbone assignment of cross-peaks is shown using the one-letter code for amino acids. '*' indicates cross-peak invisible at the plotting level. 'linker' indicates cross-peaks from residues (GPLGS) derived from the vector. Related to Figures 1, 2 and 3.

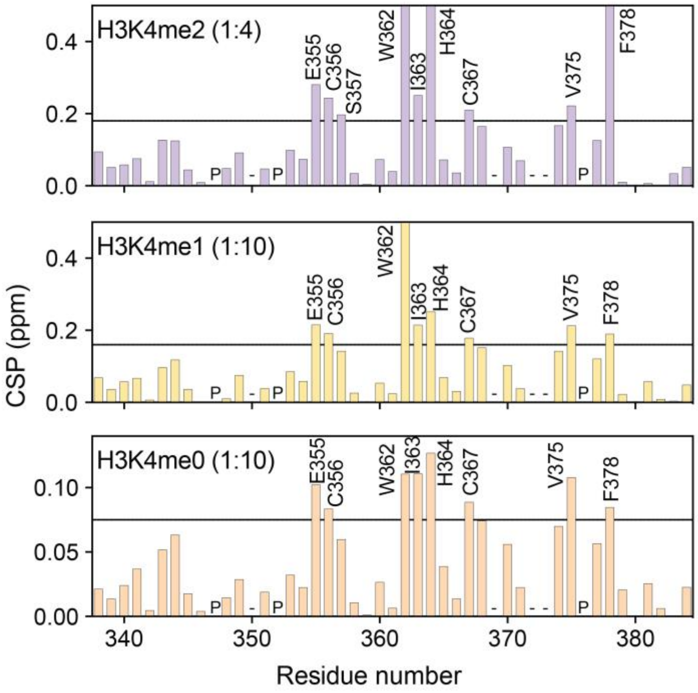

Supplementary Figure 3. Recognition of histone H3 peptides by PHF23PHD.
Chemical shift perturbation (CSP) analysis of PHF23PHD upon binding of H3K4me2 (1:4, top), H3K4me1 (1:10, middle) and H3K4me0 (1:10, bottom) peptides. 'P' indicates a proline residue. '-' indicates unassigned residue. Bars reaching the maximum of y-axis indicate disappeared cross-peaks. Related to Figures 1 and 2.

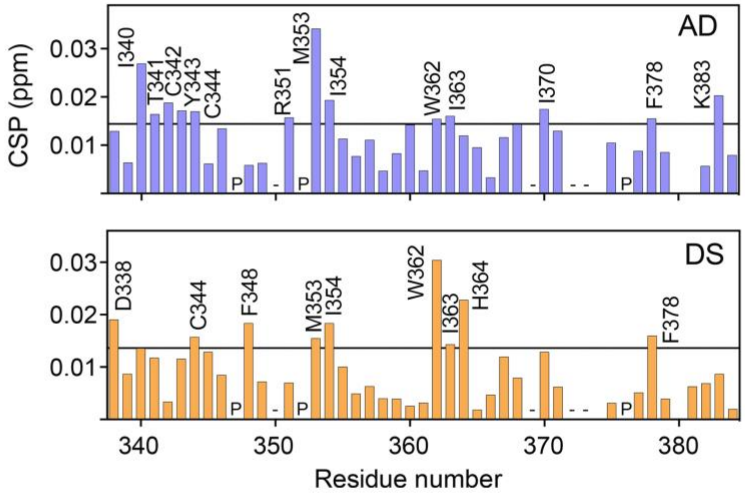

Supplementary Figure 4. Characterization of AD and DS inhibitory mechanisms. Chemical shift perturbation (CSP) analysis of the ^{1}H,^{15}N HSQC spectra of PHF23$_{PHD}$ at a 1:20 ratio of AD (purple) and 1:2 ratio of DS (orange). 'P' indicates a proline residue. '-' indicates an unassigned residue. Related to Figure 3.

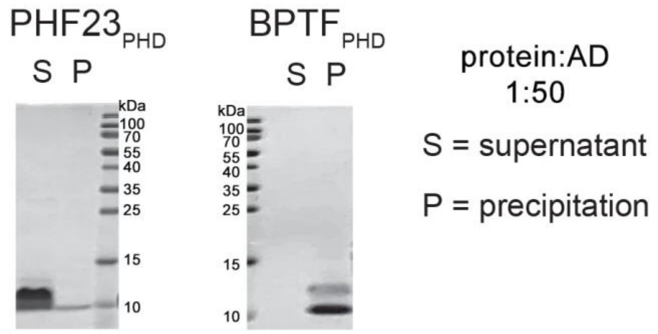

Supplementary Figure 5. Samples containing 0.1 mM $PHF23_{PHD}$ (left) and $BPTF_{PHD}$ (right) were incubated with AD at a protein:compound ratio of 1:50 and resolved by SDS-PAGE under non-reducing condition. 'S' and 'P' indicate supernatant and precipitation, respectively, after spinning the samples down. Experiment was repeated independently two times with similar results. Related to Figure 3.

a

Sample		Read # (raw)
	INPUT	9860281
	H3K4me3	8651824
	H3K27me3	13855403
NUP98-KDM5A	FLAG Rep#1	15101034
(FLAG tagged)	FLAG Rep#2	21301507

b

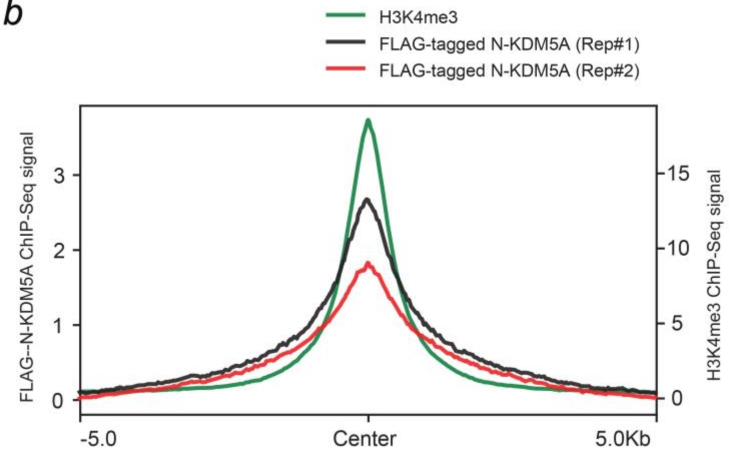

Supplementary Figure 6. NUP98-KDM5A binding sites overlap with H3K4me3-loci. (a). ChIP-seq read depths for the indicated sample. (b) Averaged H3K4me3 and NUP98-KDM5A ChIP-seq signals centered on H3K4me3 binding sites in a +/- 5.0-kb window in murine leukemia cells expressing FLAG-tagged NUP98-KDM5A. ChIP-seq signals were normalized to input. Related to Figures 5 and 6.

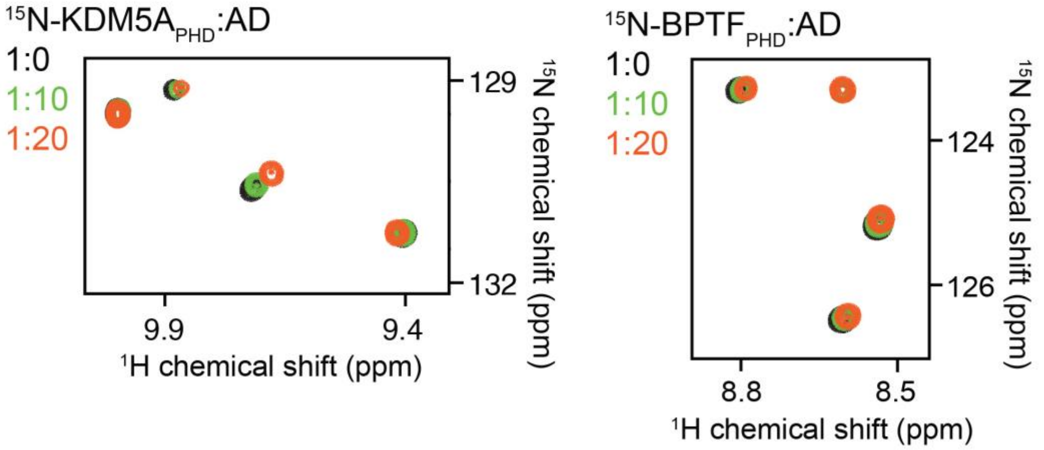

Supplementary Figure 7. AD binds to KDM5A$_{PHD}$ and BPTF$_{PHD}$. Superimposed ^{1}H,^{15}N HSQC spectra of KDM5A$_{PHD}$ (left) and BPTF$_{PHD}$ (right) collected upon titration with AD. Spectra are color coded according to the protein:compound molar ratio. Related to Figures 5 and 6.

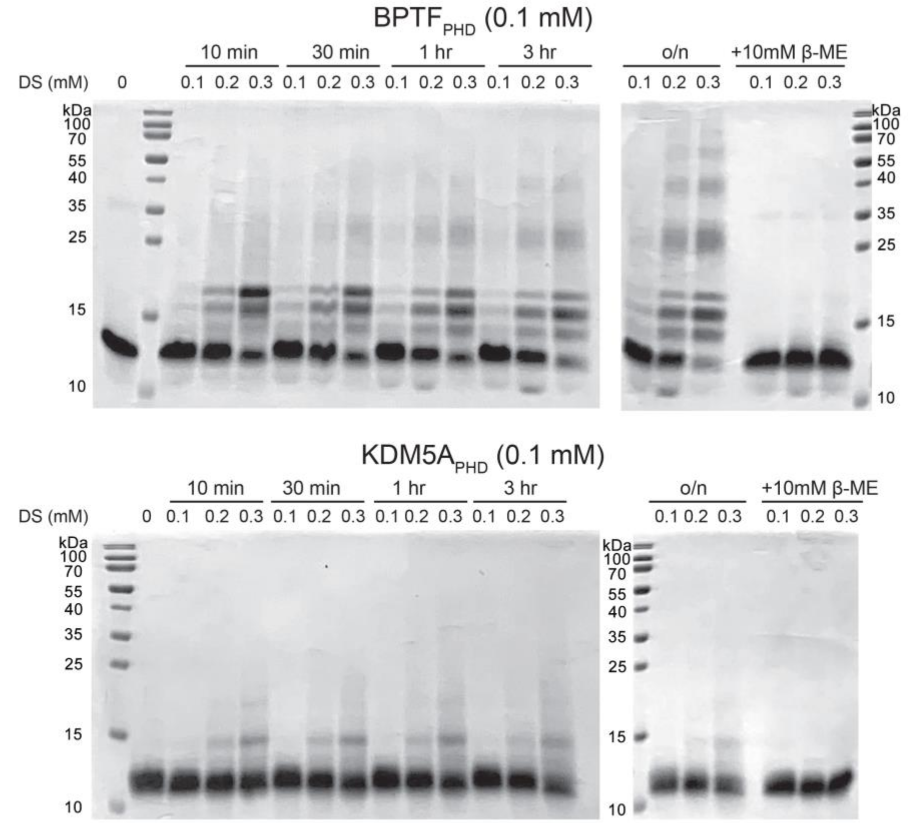

Supplementary Figure 8. Samples containing 0.1 mM BPTF$_{PHD}$ (top) and KDM5A$_{PHD}$ (bottom) were incubated with indicated amounts of DS for 10 min, 30 min, 1 hr, 3 hr, and overnight (o/n). All samples were flash-frozen and resolved by SDS-PAGE under non-reducing and reducing (10 mM β-ME) conditions. Experiment was repeated independently two times with similar results. Related to Figures 5 and 6.

Supplementary Table 1. Data collection and refinement statistics for $PHF23_{PHD}$

	**PHF23 PHD domain**
**Data Collection**	
Space group	$P\,4_1\,2_1\,2$
Wavelength (Å)	1.54
Resolution (Å)	50.0- 2.9 (3.0-2.9)*
Unit-cell dimensions	
a, b, c (Å)	66.92, 66.92, 151.05
α, β, γ (°)	90, 90, 90
No. of measured reflections	27524
No. of unique reflections	8025
Multiplicity	3.4 (3.0)
I/σ	13.4 (1.9)
Completeness (%)	97.8 (97.1)
$R_{sym\#}$ (%)	8.5 (49.4)
$R_{pim\#}$ (%)	5.1 (32.2)
No. of molecules in ASU	5
Matthews coefficient ($Å^3\,Da^{-1}$)	2.28
Solvent content (%)	46.1
**Refinement**	
R_{work}/R_{free} (%)	24.2/29.4
No. of atoms	1963
Protein	1953
Ligand/ion	10
Water	0
B-factors ($Å^2$)	58.71
Protein	58.58
Ligand/ion	84.36
R.M.S.D	
Bond lengths (Å)	0.007
Bond angles (°)	0.989
Ramachandran favored (%)	95.0
Ramachandran allowed (%)	5.0
Ramachandran outliers	0
Clashscore	8.36

*Values in parentheses are for the highest resolution shell (Å).

$^{\#}R_{sym} = \sum|I_{obs}-I_{avg}|/I_{avg}$, where I_{obs} is intensity of any given reflection and I_{avg} is the weighted mean I.

Supplementary Table 2. List of primers used in this study.

P1	CGCGAATTCGATCTGATCACATGTTACTGTCG
P2	CCGCTCGAGTTACAGTTCCTTGCATTTC
P3	CTGGGATCCGATCTGATCGCATGTTACTGTCGAAAG
P4	CTTTCGACAGTAACATGCGATCAGATCGGATCCCAG
P5	ATCACATGTTACTGTCTAAAGCCCTTTGCAGGGCGG
P6	CCGCCCTGCAAAGGGCTTTAGACAGTAACATGTGAT
P7	GTTACTGTCGAAAGCCCGCTGCAGGGCGGCCCATGAT
P8	ATCATGGGCCGCCCTGCAGCGGGCTTTCGACAGTAAC
P9	CTTTGCAGGGCGGCCCGTGATTGAGTGCAGCCTGTGT
P10	ACACAGGCTGCACTCAATCACGGGCCGCCCTGCAAAG
P11	GCCCATGATTGAGTGCAGCATGTGTGGGACGTGGATTC
P12	GAATCCACGTCCCACACATGCTGCACTCAATCATGGGC
P13	CAGCCTGTGTGGGACGGCGATTCACCTCTCCTGTGCT
P14	AGCACAGGAGAGGTGAATCGCCGTCCCACACAGGCTG
