# Supplementary material for: Mechanistic insights into chromatin targeting by leukemic NUP98-PHF23 fusion
Source: Nat Commun. 2020 Jul 3;11:3339. doi: 10.1038/s41467-020-17098-4 (PMC7335091; doi:10.1038/s41467-020-17098-4)
Supplement: Supplementary file 3 — Reporting Summary [file 41467_2020_17098_MOESM3_ESM.pdf]

nature research

Corresponding author(s): Tatiana Kutateladze

Reporting Summary

Nature Research wishes to improve the reproducibility of the work that we publish. This form provides structure for consistency and transparency in reporting. For further information on Nature Research policies, see Authors & Referees and the Editorial Policy Checklist.

Statistical parameters

When statistical analyses are reported, confirm that the following items are present in the relevant location (e.g. figure legend, table legend, main text, or Methods section).

n/a	Confirmed	
	x	The exact sample size (n) for each experimental group/condition, given as a discrete number and unit of measurement
	x	An indication of whether measurements were taken from distinct samples or whether the same sample was measured repeatedly
x		The statistical test(s) used AND whether they are one- or two-sided<br>*Only common tests should be described solely by name; describe more complex techniques in the Methods section.*
x		A description of all covariates tested
x		A description of any assumptions or corrections, such as tests of normality and adjustment for multiple comparisons
x		A full description of the statistics including central tendency (e.g. means) or other basic estimates (e.g. regression coefficient) AND variation (e.g. standard deviation) or associated estimates of uncertainty (e.g. confidence intervals)
x		For null hypothesis testing, the test statistic (e.g. F, t, r) with confidence intervals, effect sizes, degrees of freedom and P value noted<br>*Give P values as exact values whenever suitable.*
x		For Bayesian analysis, information on the choice of priors and Markov chain Monte Carlo settings
x		For hierarchical and complex designs, identification of the appropriate level for tests and full reporting of outcomes
x		Estimates of effect sizes (e.g. Cohen's d, Pearson's r), indicating how they were calculated
	x	Clearly defined error bars<br>*State explicitly what error bars represent (e.g. SD, SE, CI)*

Our web collection on statistics for biologists may be useful.

Software and code

Policy information about availability of computer code

Data collection	NMR experiments were carried out at 298K on Varian INOVA 500 and Bruker 600 MHz spectrometers at the UC Denver NMR Core facility. X-ray diffraction data were collected at 100K at the UC Denver X-ray Crystallography core facility.
Data analysis	NMRPipe, CcpNmr Suite v2.1, I-PINE web server (http://i-pine.nmrfam.wisc.edu), COOT 0.8.2, and PHENIX (with MOLProbity) v1.6<br>ChIP-seq peak calling: Trim galore version 0.6.1 was used for adapter trimming and quality filter for all reads. STAR v2.7.1a was used to align the reads to the mouse genome (mm9). Samtools (v1.9), Picard MarkDuplicates funtion (ver 2.20.4), and bedtools (v2.28.0) were used to remove the non-primary alignment, PCR duplicates, or blacklist regions from aligned data. MACS2(v2.1.1) was used for peak calling. Deeptools (v3.3.0) was used to make bigwig files, heatmaps, and averaged plottings of ChIP-seq signal. These bigwig files were visualized using IGV v2.5.3.

For manuscripts utilizing custom algorithms or software that are central to the research but not yet described in published literature, software must be made available to editors/reviewers upon request. We strongly encourage code deposition in a community repository (e.g. GitHub). See the Nature Research guidelines for submitting code & software for further information.

Data

Policy information about availability of data

All manuscripts must include a data availability statement. This statement should provide the following information, where applicable:

- Accession codes, unique identifiers, or web links for publicly available datasets
- A list of figures that have associated raw data
- A description of any restrictions on data availability

Coordinates and structure factors for PHF23-PHD have been deposited in the Protein Data Bank (PDB ID 6WXK). The ChIP-seq data is submitted to GEO under the accession number GSE146693. All other relevant data supporting the key findings of this study are available within the article and its Supplementary Information files or from the corresponding authors upon reasonable request.

Field-specific reporting

Please select the best fit for your research. If you are not sure, read the appropriate sections before making your selection.

☒ Life sciences ☐ Behavioural & social sciences ☐ Ecological, evolutionary & environmental sciences

For a reference copy of the document with all sections, see nature.com/authors/policies/ReportingSummary-flat.pdf

Life sciences study design

All studies must disclose on these points even when the disclosure is negative.

Sample size	Sample sizes of 3 independent experiments was used unless otherwise stated. This is noted in each individual figure legend. For most experiments, no statistical size calculation was performed as it was impossible to estimate the effect in advance. In most of our described experiments, the effect was obvious, the sample conditions differed clearly from control conditions and the reported independent repeats were sufficient to show this significant effect.
Data exclusions	no data exclusions
Replication	Independent repeats were performed as stated in each figure legend. All attempts at replication were successful.
Randomization	All experiments were performed in vitro and randomization was not applicable as our study did not involve clinical trials.
Blinding	No blinding was used in these in vitro experiments. This study did not involve clinical trials.

Reporting for specific materials, systems and methods

Materials & experimental systems

n/a	Involved in the study
☒	☐ Unique biological materials
☐	☒ Antibodies
☐	☒ Eukaryotic cell lines
☒	☐ Palaeontology
☐	☒ Animals and other organisms
☒	☐ Human research participants

Methods

n/a	Involved in the study
☐	☒ ChIP-seq
☒	☐ Flow cytometry
☒	☐ MRI-based neuroimaging

Antibodies

Antibodies used

anti-GST (sc-459) WB 1/2000
"Mouse anti-Flag tag (M2) Sigma F1804" WB 1/1000
"Mouse anti-Flag tag (M2) Sigma F1804" ChIP 5 ug used for ChIP
tri-methylated H3K4 (H3K4me3); abcam ab9050 ChIP 5 ug used for ChIP
tri-methylated H3K27 (H3K27me3); millipore 07-449 ChIP 5 ug used for ChIP

Validation

All antibodies used in this study are commercially available. Antibodies were validated by the manufacturers and used in the following studies:
Jeschke, A. et al. 2017. Methods in enzymology. 587: 271-291.
Anantharaman, Aparna et al. Nucleic acids research vol. 45,7 (2017): 4189-4201.
Schumacher J et al. Fungal Genet Biol 123:14-24 (2019).
Gao R, Chen S, Kobayashi M, et al. Stem Cells. 2015;33(3):925-938.

Eukaryotic cell lines

Policy information about cell lines

Cell line source(s)

Immortal, cytokine independent leukemic cell lines that express a NUP98-PHF23 fusion (748T and 961C) were established from NUP98-PHF23 transgenic mice9. 748T is a precursor T cell line and 961C is a myeloid cell line. 188G3 and 189E6 are IL3-dependent cell lines established from embryonic stem cells that express a NUP98-HOXD13 fusion from the endogenous NUP98 locus33. 7298/2 is a precursor T cell line established from SCL/LMO1 double transgenic mice34. 32D is an IL-3 dependent, spontaneously immortalized, murine myeloid cell line35. Murine AML lines transformed by NUP98-KDM5A was generated and maintained as previously described8. All cell lines were maintained in Iscove's modified Dulbecco's medium (IMDM) supplemented with 15-20% fetal bovine serum (FBS), 100 mM L-glutamine and 100 µg/mL penicilin/streptomycin (Invitrogen). 188G3, 189E6, and 32D cells were also supplemented with IL-3 (10 ng/mL) (Peprotech).
Primary bone marrow cells are harvested from femur and tibia of wild-type balb/C mice and then subject to a lineage-negative (Lin-) enrichment protocol to remove differentiated cell populations as described8. Such Lin- enriched HSPCs were first stimulated in the base medium (OptiMEM, Invitrogen, cat#31985), 15% of FBS (Invitrogen, cat#16000-044), 1% of antibiotics and 50 µM of β-mercaptoethanol) complemented with a cytokine cocktail that contains 10 ng/mL each of murine SCF (Peprotech), Flt3 ligand (Flt3L; Sigma), IL3 (Peprotech) and IL6 (Peprotech) for 4 days. MSCV based retrovirus encoding the NUP98-PHD finger fusion oncogene was produced in 293 cells. Two days post-infection with retrovirus, murine HSPCs were subject to drug selection and then plated for assaying proliferation and differentiation in the same liquid base medium with SCF alone as described8. These in vitro cultured HSPC cells were routinely monitored under microscopy and cellular morphology examined by Wright-Giemsa staining.

Authentication

Authentication of the cell lines is not relevant since they were generated in the lab.

Mycoplasma contamination

Cell lines were not tested for mycoplasma contamination.

Commonly misidentified lines
(See ICLAC register)

No commonly misidentified cell lines were used in the study.

Animals and other organisms

Policy information about studies involving animals; ARRIVE guidelines recommended for reporting animal research

Laboratory animals

No animals were used in this study. Only cells and cell lines derived from transgenic mice established previously were used.
Wang, G. G. et al. Haematopoietic malignancies caused by dysregulation of a chromatin-binding PHD finger. Nature 459, 847-851, (2009).
Gough, S. M. et al. NUP98-PHF23 is a chromatin-modifying oncoprotein that causes a wide array of leukemias sensitive to inhibition of PHD histone reader function. Cancer discovery 4, 564-577, (2014).

Wild animals

No wild animals were used in this study.

Field-collected samples

No field-collected samples were used in this study.

ChIP-seq

Data deposition

- [x] Confirm that both raw and final processed data have been deposited in a public database such as GEO.
- [x] Confirm that you have deposited or provided access to graph files (e.g. BED files) for the called peaks.

Data access links
May remain private before publication.

https://www.ncbi.nlm.nih.gov/geo/query/acc.cgi?acc=GSE146693

Files in database submission	GSM4404139 NUP98-KDM5A_AML Input<br>GSM4404140 NUP98-KDM5A_AML N5A ChIP replicate 1<br>GSM4404141 NUP98-KDM5A_AML N5A ChIP replicate 2<br>GSM4404142 NUP98-KDM5A_AML H3K4me3 ChIP<br>GSM4404143 NUP98-KDM5A_AML H3K27me3 ChIP
Genome browser session (e.g. UCSC)	N/A.

Methodology

Replicates	ChIP-Seq data are from two experiments.
Sequencing depth	ChIP-seq depth was described in Supplementary information provided.
Antibodies	"Mouse anti-Flag tag (M2) Sigma F1804" ChIP 5 ug used for ChIP<br>tri-methylated H3K4 (H3K4me3); abcam ab9050 ChIP 5 ug used for ChIP<br>tri-methylated H3K27 (H3K27me3); millipore 07-449 ChIP 5 ug used for ChIP
Peak calling parameters	ChIP-seq was carried out and ChIP-seq data alignment, filtration, peak calling and assignment, and cross-sample comparison were performed as described36.
Data quality	ChIP-seq was carried out and ChIP-seq data alignment, filtration, peak calling and assignment, and cross-sample comparison were performed as described36.
Software	ChIP-seq peak calling: Trim galore version 0.6.1 was used for adapter trimming and quality filter for all reads. STAR v2.7.1a was used to align the reads to the mouse genome (mm9). Samtools (v1.9), Picard MarkDuplicates funtion (ver 2.20.4), and bedtools (v2.28.0) were used to remove the non-primary alignment, PCR duplicates, or blacklist regions from aligned data. MACS2(v2.1.1) was used for peak calling. Deeptools (v3.3.0) was used to make bigwig files, heatmaps, and averaged plottings of ChIP-seq signal. These bigwig files were visualized using IGV v2.5.3.
